# Topically applied bacteriophage to control multi-drug resistant *Pseudomonas aeruginosa*-infected wounds in a New Zealand rabbit model

**DOI:** 10.3389/fmicb.2022.1031101

**Published:** 2022-10-18

**Authors:** Jinyu Wang, Wenxin Meng, Kaichuan Zhang, Jingyu Wang, Baochun Lu, Ruijie Wang, Kun Jia

**Affiliations:** Guangdong Provincial Key Laboratory of Comprehensive Prevention and Control for Severe Clinical Animal Diseases, College of Veterinary Medicine, South China Agricultural University, Guangzhou, China

**Keywords:** *Pseudomonas aeruginosa*, multi-drug resistant, phage therapy, phage cocktail, characterization

## Abstract

*Pseudomonas aeruginosa* (*P. aeruginosa*) is a widespread, gram-negative, pathogenic bacterium that causes serious internal and external infections in humans and other animals. The increasing antibiotic resistance has complicated bacterial infection treatment, and current antibiotic therapies cannot cure all infections. Owing to this, bacteriophages (phages) have regained attention as potential therapeutics for bacterial infections. In this study, the phage “PaVOA” was isolated from hospital sewage and characterized. Next, a New Zealand rabbit skin infection model was used to determine the therapeutic efficacy of PaVOA as compared to a phage cocktail or the cephalosporin antibiotic ceftriaxone. Characterization results demonstrated that phage PaVOA belongs to the *Myoviridae* family, has a double-stranded DNA genome, is resistant to low temperatures (−20°C), is most optimal at 40°C, has good acid–base tolerance, and remains stable for 30 min under 20 W ultraviolet (UV) intensity. The optimal multiplicity of infection of PaVOA was 0.1, and a one-step growth curve showed a short latency period (10 min), thus demonstrating its ability to rapidly kill bacteria. Furthermore, the addition of calcium (Ca) and magnesium (Mg) ions significantly increased the PaVOA titer. An *in vivo* phage kinetic curve showed that PaVOA was rapidly inactivated within the blood of New Zealand rabbits (undetectable after 12 h), and no animals died due to phage treatment. Wound healing studies showed that the phage cocktail induced a high healing rate and an acceleration of the skin remodeling process, and was more efficacious than ceftriaxone. Therefore, phage cocktail therapy represents a novel therapeutic approach in the treatment of traumatic skin infections caused by multi-drug resistant *P. aeruginosa*.

## Introduction

*Pseudomonas aeruginosa* (*P. aeruginosa*), a gram-negative bacterium, is a common opportunistic pathogen in hospitals that can cause serious infections such as pneumonia, urinary tract infections, burn infections, and bacteremia (Moradali et al., 2017). Importantly, *P. aeruginosa* accounts for 10%–20% of all bacterial cases of ventilator-associated pneumonia (Weiner et al., 2016) and is one of the leading causes of urinary tract infections (Reynolds and Kollef, 2021). Patients with burns are more likely to be infected by *P. aeruginosa* and suffer from complications in a humid environment (Norbury et al., 2016), whilst the mortality rate of *P. aeruginosa* bacteremia is as high as 43.2%–58.8% (Thaden et al., 2017).

Antibiotic treatment for infections caused by *P. aeruginosa* and infection control is hampered by its ubiquity in the environment and its intrinsic drug resistance (Breidenstein et al., 2011; Poole, 2011). Additionally, the prevalence of multi-drug resistant (MDR) *P. aeruginosa* is increasing. In recent years, bacterial infections in Spain and the United States have had a prevalence rate of 15%–30% (Pena et al., 2015; Sader et al., 2018). Drug-resistant bacterial infections are becoming an urgent issue, with the WHO reporting that 10 million people will die per year by 2050 from drug-resistant bacterial infections (Taati Moghadam et al., 2020). Therefore, novel approaches are urgently required to treat drug-resistant bacterial infections.

Bacteriophages (phages) are viruses that are small in size, do not have a complete cellular structure, and contain only a single nucleic acid. Phages have an estimated population of over 10^31^ on Earth (Dion et al., 2020) and can be isolated from soil, seawater, sewage, and waste products. Moreover, the mechanism whereby phages kill bacteria is unique and has several advantages; they have high host specificity, are non-toxic to humans and animals, and have minimal effects on nonspecific flora (Yoon Kyung Chang et al., 2022). Importantly, phages are considered a potential alternative to antibiotics for treating bacterial infections because of their high adaptability and efficacy against MDR pathogens in various environments (Alsaadi et al., 2021; Chegini et al., 2021). The application of phages for treating MDR bacterial infections is becoming more common for treating conditions such as infected skin burns, surgical wounds, and trauma wounds. A recent animal study showed that phage therapy for wounds infected with MDR *Klebsiella pneumoniae* resulted in the highest healing efficiency observed in the study (Fayez et al., 2021), thus supporting the potential therapeutic application of phage therapies.

The human skin functions to protect underlying tissues, muscles, and organs from physical and chemical damage (Bowler et al., 2001). However, direct contact with the external environment predisposes the skin to damage, resulting in a risk of sepsis due to microbial infiltration into wounds caused by burns, surgery, or trauma (Stearns-Kurosawa et al., 2011). *Pseudomonas aeruginosa* is one of the most prevalent causative pathogens of wound infections in hospitals (Chua et al., 2016). The main component of the bacterial cell wall, lipopolysaccharide, is highly immunogenic to infected patients, and the presence of MDR *P. aeruginosa* aggravates infection and hinders treatment.

For modest skin infections, the conventional methods of treatment include debridement, washing with a large amount of saline, and iodophor disinfection. In severe cases, hydrogen peroxide may be used either alone or in combination with antibiotics to enhance the therapeutic effect; however, the presence of MDR bacteria must be considered in such cases. Moreover, topical application of probiotics can reduce bacterial infections and promote wound healing (Fijan et al., 2019). However, as the structure and function of the skin microbiome are complex, the therapeutic effect of probiotics cannot be guaranteed (Krezalek and Alverdy, 2018). Due to these issues, phage therapy has received renewed attention in recent years.

As a model organism, the New Zealand rabbit has desirable characteristics of large serum volume, sensitivity to body temperature changes, and skin sensitivity to most pathogenic bacteria. Owing to these characteristics, they are commonly used experimentally for pyrogenic, immunological, and pharmacological studies. Malachowa (Malachowa et al., 2022), among others, demonstrated the successful optimization of *Staphylococcus aureus* skin infection in rabbits, which provides a reference for the establishment of other skin infection models. The present study aimed to characterize the laboratory-isolated *P. aeruginosa* phage PaVOA and subsequently evaluate its efficacy in resolving *P. aeruginosa*-mediated wound infections in New Zealand rabbits.

## Materials and methods

### PaVOB

PaVOB was sourced from the Department of Surgery, School of Veterinary Medicine, South China Agricultural University, China.

### Ethics statement

Animal experiments were carried out according to the regulations of the ethics committee of South China Agricultural University. All animal experiments complied with the guidelines of the China Animal Welfare Commission.

### Bacterial strains and culture conditions

Twenty-eight clinically isolated *P. aeruginosa* strains from the Department of Surgery, College of Veterinary Medicine, South China Agricultural University were included in this study. A standard *P. aeruginosa* strain CMCC 10104 was used as a reference strain. All strains were grown in LB broth (Qingdao high tech Industrial Park Haibo Biotechnology Co., Ltd. Qingdao, China) and cultured on a shaking table at 37°C for 12 h. Bacterial DNA was isolated using the TIANamp Bacteria DNA Kit (Tiangen Biotech, Beijing, China). All strains were identified as *P. aeruginosa* by PCR experiments.

#### Antibiotic sensitivity test

We referred to the recommendations for *P. aeruginosa* drug susceptibility testing provided by the National Committee for Clinical Laboratory Standards (NCCLS) for drug selection. A total of 22 antibiotics (Hangzhou Binhe microbial Reagent Co., Ltd. Hangzhou, China), including ciprofloxacin (CIP; 5 μg), amikacin (AK; 30 μg), ceftriaxone (CRO; 30 μg), meropenem (MEM; 10 μg), gentamicin (CN; 10 μg), doxycycline (DO; 30 μg), imipenem (IPM; 10 μg), amoxicillin (AM; 20 μg), cefepime (CPM;30 μg), ofloxacin (OF;5 μg), compound sulfamethoxazole (SXT; 15 μg), enrofloxacin (ENR; 5 μg), ampicillin (AMP; 10 μg), azithromycin (AZM; 15 μg), chloramphenicol (C; 10 μg), tetracycline (TE; 30 μg), nincomycin (MY; 10 μg), norfloxacin (NOR; 10 μg), ceftazidime (CAZ; 30 μg), mezlocillin (MEZ; 30 μg), cefoperazone (CFP; 30 μg), and tobramycin (TOB; 10 μg) were used for the antibacterial sensitivity testing of 29 *P. aeruginosa* strains using the paper diffusion method. The diameter of inhibition circles was used to evaluate the sensitivity of *P. aeruginosa* to all antibiotics tested.

### Phage isolation, purification, and host range determination

Two different bacteriophages were isolated from the sewage of Nanfang Hospital of the Southern Medical University of China. For water sampling, a 0.22 μM filter membrane (Qingdao high tech Industrial Park Haibo Biotechnology Co., Ltd. Qingdao, China) was used to remove residual bacteria, followed by centrifugation of the filtered filtrate at 12,000 rpm for 15 min at 25°C, with the resulting supernatant enriched with *P. aeruginosa*. The double-layer agar plate method was then used to separate phages. Individual phage plaques were chosen for purification once their size and morphology were consistent (purified single phages). One of the lytic phages with transparent plaques was selected, and *P. aeruginosa* (PA/18) was used as the host bacteria to further study the biological characteristics of the selected phages. Moreover, the host profile of the phage was assessed using the spotting method.

#### Transmission electron microscopy of phage particles

Purified phage particles with a titer of 10^11^ PFU/mL were loaded onto a copper grid for 1 min, followed by negative staining with uranyl acetate (2% w/v) for 1 min and subsequent aspiration of excess liquid. After drying, the Talos™ F200S TEM (Thermo Fisher Scientific, Waltham, MA, USA) apparatus was used to observe the morphology of phages and to capture images.

#### Phage nucleic acid type identification

Purified phage concentrates were prepared using the PEG precipitation extraction method (Rathor et al., 2022). Phage genomic DNA was extracted using the Viral DNA Kit (OmegaBiotek, Norcross US). Isolated DNA was treated with DNase I (20 U/μg; Solarbio Science & Technology Co., Ltd., Beijing, China), RNase A (5 U/μg; Solarbio Science & Technology Co., Ltd., Beijing, China), and Mung Bean Nuclease (20 U/μg; TaKaRa Biomedical Technology Co., Ltd., Beijing, China) and incubated at 37°C for enzymatic digestion of the genome. The resulting samples were separated by 0.8% (w/v) agarose gel electrophoresis.

### Phage biological characteristics

#### Determination of optimal multiplicity of infection

*Pseudomonas aeruginosa* was cultured in LB broth for 16–18 h. Overnight cultures were then diluted with LB broth to a concentration of 10^8^ CFU/ml. Phages were added at MOIs of 1 × 10^−4^–1 × 10^2^ and incubated for 4 h at 37°C in a warm oven after shaking and mixing. Cocktails were centrifuged at 12,000 rpm for 10 min followed by isolation of the supernatant to determine phage titers using the double-layer agar plate method. The experiment was repeated thrice.

#### One-step growth curve

*Pseudomonas aeruginosa* was cultured to the logarithmic growth phase, at which point the PaVOA phage was added according to the optimal MOI ratio determined previously. Cultures were placed in a 37°C incubator for 15 min prior to centrifugation at 8,000 rpm for 5 min. After discarding the supernatant, sediment was resuspended with preheated sterile LB broth. The suspension cocktail was cultured on a shaking table at 37°C, 200 rpm. Samples were taken at intervals of 10 min in the first 30 min and then every 15 min. Phage titers of samples were determined using the double-layer agar plate method. The experiment was repeated thrice, and one-step growth curves were plotted using GraphPad Prism 8 (San Diego, CA, USA) software. The burst size of the phage is equal to the amount of phage at the end of lysis divided by the initial number of bacterial cells at the time of infection (Xu et al., 2021).

#### Optimal phage temperature, pH, and UV stability

Aliquots of PaVOA phages with determined titers were incubated in water baths at a range of temperatures (−20°C, 4°C, 25°C, 37°C, 40°C, 50°C, 60°C, 70°C, and 80°C) for 1 h. Three parallel controls were set up for each temperature, and titers were measured using the double-layer agar plate method. Additionally, the PaVOA phage was exposed to sterile Petri dishes at a distance of 40 cm from UV light (20 W) for 1 h, and samples were taken at 5 and 10 min intervals to determine phage stability. The titers of PaVOA phages were determined using the double-layer agar plate method at a pH range from 1 to 14, with pH conditions controlled using SM buffer. Experiments were repeated thrice.

#### Effect of calcium and magnesium ions on phage activity

Host bacteria (PA/18) were inoculated in LB, LB-Ca, LB-Mg, and LB-Ca-Mg liquid medium (all ion concentrations were 0.1 mol/l) and then cultured in a shaking table at 37°C, 200 rpm until the OD600 was approximately 0.3. PaVOA phages were added at the optimal MOI as previously determined and cultured for 3 h. The resulting supernatants were collected by centrifugation at 12,000 rpm for 15 min at 4°C, with phage titers being determined by the double-layer agar plate method.

#### Phage inactivation test in New Zealand rabbits

New Zealand rabbits (3 months old) were purchased from Guangdong Provincial Medical Laboratory Animal Center (Guangzhou, China). Purified PaVOA phage samples were diluted to a concentration of 10^9^ PFU/mL with normal saline. New Zealand rabbits underwent shaving and disinfection of their ears, followed by slow injections of 1 ml diluted phage preparations through the ear marginal vein. Volumes of 500 μl whole blood were collected into anticoagulation tubes at different points (5 min, 15 min, 30 min, 60 min, 3 h, 6 h, and 24 h) throughout the experiment. Whole blood samples were centrifuged at 12,000 rpm for 10 min at 4°C with the resulting supernatants being diluted in PBS. Phage titers at each time point were determined by the double-layer agar plate method to evaluate phage survival rate.

### Comparison of wound healing efficiency between PaVOA phage and ceftriaxone on *Pseudomonas aeruginosa*-infected wounds in a New Zealand rabbit model

#### Surgical procedures for full-thickness wound models

The efficacy of phages in the treatment of traumatic skin bacterial infection was evaluated using phages and antibiotics to treat full-thickness skin excision wounds of New Zealand rabbits inoculated with *P. aeruginosa*. Twenty-five New Zealand rabbits (3 months old) weighing approximately 1.9–2.1 kg were housed in sterilized cages and provided with adequate water and rabbit food. After 7 days of adaptive feeding, New Zealand rabbits were depilated at approximately 2 cm on the left side of the spine of their back, followed by local anesthesia using 0.5% lidocaine. Surgical scissors were then used to create square allograft wounds with a side length of approximately 2 cm (Shalaby et al., 2019; Kifelew et al., 2020). Wound infections were induced by inoculation of a 10^8^ CFU/ml *P. aeruginosa* bacterial solution (PA/18) by syringe, after which wounds were covered with sterile dressings and adhesive bandages. Successful replication of the previously described model should result in purulent secretion on the wound surface, mild tissue erythema and edema, and slightly elevated skin temperature.

Following the successful replication of the wound infection model, New Zealand rabbits were randomly divided into five groups: negative control (infection-free), positive control (infection), and three treatment groups (phage alone treatment, phage cocktail treatment, and ceftriaxone treatment), with five animals in each group. The administration methods for each group were as follows: negative and positive control groups were treated with 0.9% NaCl dropped onto their sterile gauzes, whilst single bacteriophage (PaVOA, 10^8^ PFU/mL), bacteriophage cocktail (PaVOA and PaVOB, 10^8^ PFU/mL), and ceftriaxone groups (0.002%) received 2 ml of relevant solutions onto their sterile gauzes. Sterile adhesive dressings were then used to cover and fix all gauzes, followed by 4 days of treatment. Wound healing effects were evaluated at fixed points (days 0, 3, 7, 12, 17, and 22) using a digital camera and Vernier Calipers. Wound healing (%) was calculated using the following equation:


$$\begin{array}{l} \mathrm{Wound healing}\;\left(\%\right)\\ =\left[\begin{array}{l} \left(\mathrm{wound area}\;\mathrm{at}\;\mathrm{time point tested}-\mathrm{wound area}\;\mathrm{at}\;\mathrm{day}\;0\right)\\ /\mathrm{wound area}\;\mathrm{at}\;\mathrm{day}\;0 \end{array}\right]\\ \;\times 100 \end{array}$$

#### Histological examination

Tissues for histological examination were obtained from wound edges on days 0, 3, 7, and 22. The isolated tissues were fixed in a 4% formaldehyde solution and were subsequently embedded in paraffin. Fixed sections were stained with hematoxylin and eosin (H&E).

#### Statistical analysis

All experimental data were collected and organized using Microsoft Excel and then plotted and analyzed using GraphPad Prism 8. Data are presented as mean ± standard deviation (SD).

## Results

### Antibiotic sensitivity profile

The drug sensitivity results of the 22 antibiotics tested on 29 strains of *P. aeruginosa* showed that all strains were highly resistant to cotrimoxazole, amoxicillin, and lincomycin. Additionally, more than 75% of the strains were highly sensitive to amikacin, ceftriaxone, cefepime, meropenem, ofloxacin, gentamicin, imipenem, azithromycin, tetracycline, ceftazidime, mezlocillin, cefoperazone, and tobramycin. The remaining 25% showed no obvious regularity concerning antibiotic resistance. Additional data showed that the host strain PA/18 was resistant to six antibiotics and was an MDR strain.

### Phage studies

#### Phage morphology and host range identification

**Figure 1:**
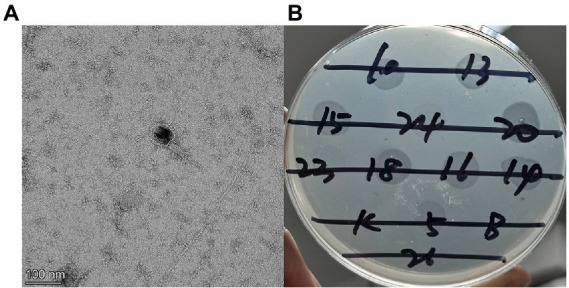
Transmission electron microscopy **(A)** and host range **(B)** of phage PaVOA.

TEM showed that each PaVOA virus particle had an icosahedral head with a diameter of 66 ± 1 nm and a contracted tail with a length of 120 ± 7 nm (Figure 1A), which is a typical morphological feature of the *Myoviridae* family. Additionally, the host spectrum of PaVOA was determined by the spotting method, and the cleavage rate reached 8/29, indicating that the host spectrum of PaVOA was narrow.

#### Phage nucleic acid type identification

The results of nuclease-mediated digestion of the phage genome revealed no bands in the DNase I treatment group, whilst the size and position of the bands in the negative control, RNase A, and the Mung Bean Nuclease treatment groups were effectively identical, indicating that the PaVOA genome is only sensitive to DNase I, thus proving that PaVOA possesses a double-stranded DNA (dsDNA) genome.

#### Optimal MOI and one-step growth curve

**Figure 2:**
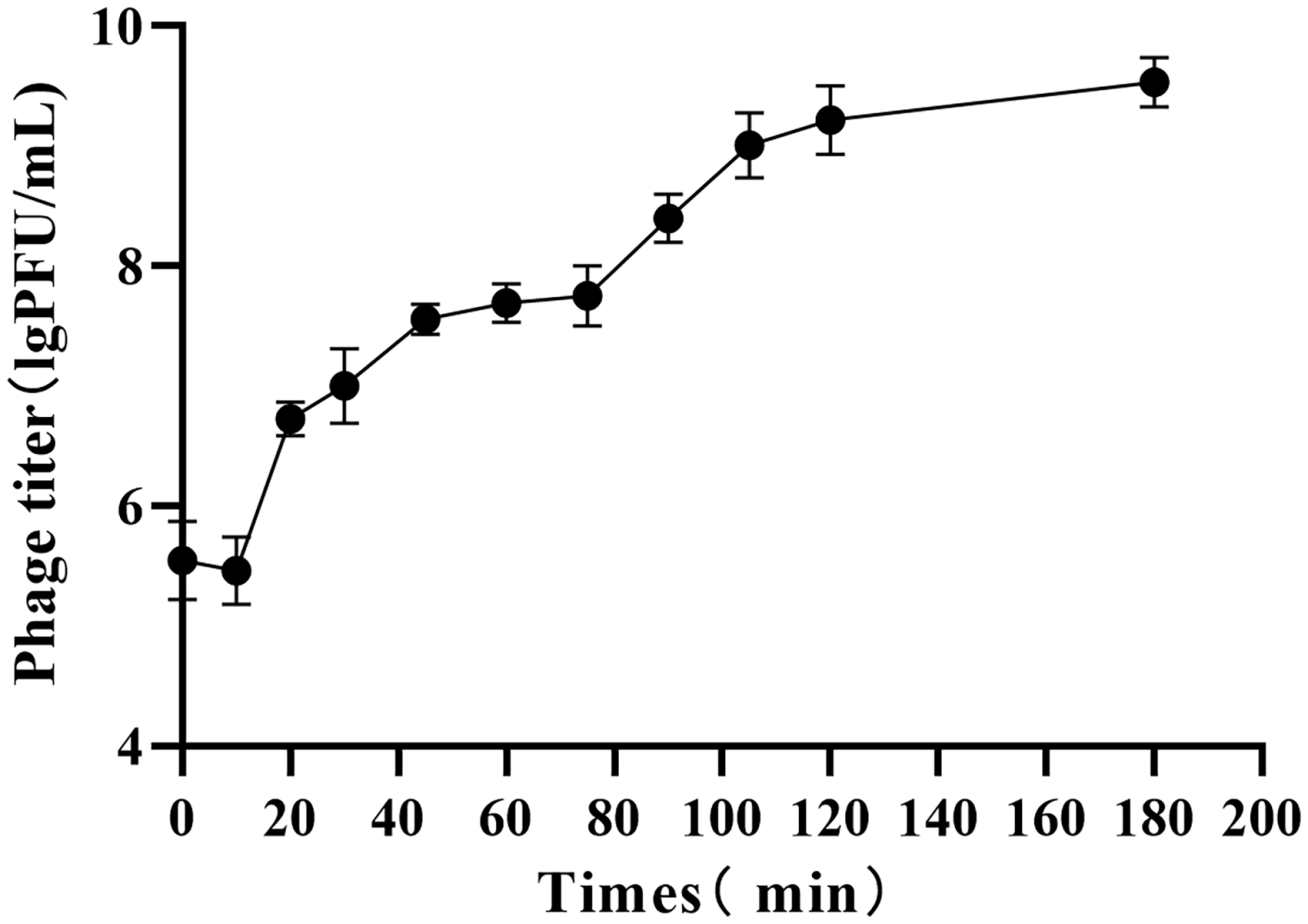
One-step growth curve of phage PaVOA.

An MOI of 0.1 resulted in the largest phage lysate titer (Table 1). The results of the one-step growth curve established according to the optimal MOI of 0.1 showed an upward trend of the PaVOA titer after 10 min, which tended to plateau at 105 min (Figure 2). Therefore, the incubation period of phage PaVOA is 10 min, and the lysis period is 20 min. According to the formula shown below, the burst amount was found to be 154 PFU/cell.

**Table 1:** Determining the optimal multiplicity of infection of phage PaVOA.

MOI	PFU of phage PaVOA	CFU of *Pseudomonas aeruginosa* 18	Phage PaVOA titers (PFU/ml)
100	1×10^9^	1×10^7^	4.1×10^8^
10	1×10^9^	1×10^8^	3.95×10^8^
1	1×10^8^	1×10^8^	4.2×10^8^
0.1	1×10^7^	1×10^8^	3.2×10^9^
0.01	1×10^6^	1×10^8^	1.89×10^9^
0.001	1×10^5^	1×10^8^	2.1×10^9^
0.0001	1×10^4^	1×10^8^	2.23×10^9^

#### Optimal phage temperature, pH, and UV stability

**Figure 3:**
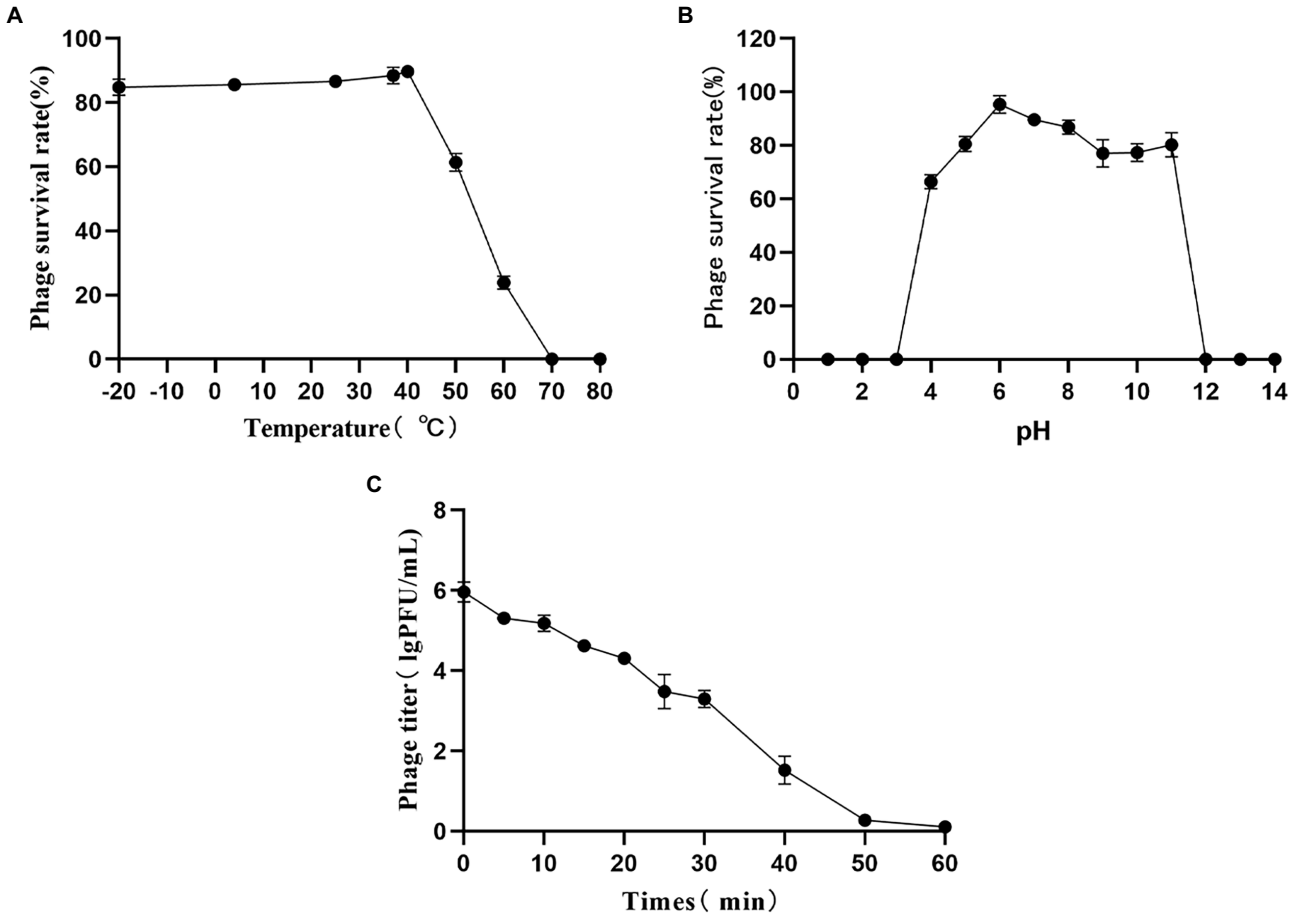
Temperature **(A)**, pH **(B)**, and UV **(C)** stability of phage PaVOA.

The results of stability tests of PaVOA showed that the phage was able to tolerate low temperatures, and the survival rate did not change significantly between −20°C and 4°C. The highest survival rate was observed at 40°C. Decreasing survival was observed at temperatures above 40°C, and most notably at 70°C (Figure 3A). The phage survival rate was greater than 60% between pH 4–11, and the survival rate was close to 100% at pH 6, indicating that PaVOA has a good acid–base tolerance (Figure 3B). Additionally, the stability of PaVOA under 20 W ultraviolet light was studied. The results showed that PaVOA was relatively stable in the first 30 min, whereas the titer decreased sharply after 30 min, but the phage did not completely lose its activity until 60 min (Figure 3C).

#### Effect of calcium and magnesium ions on phage activity

**Figure 4:**
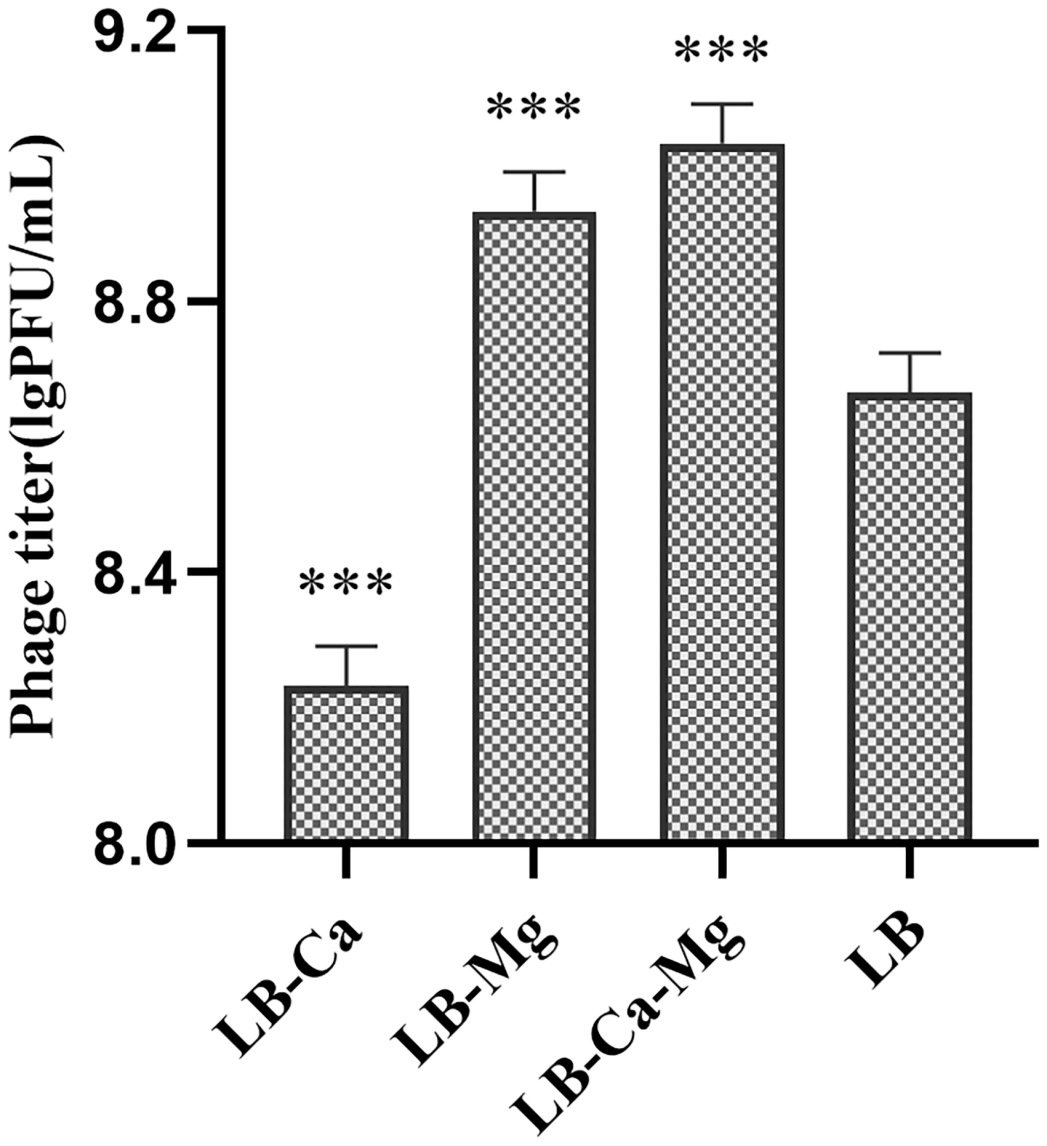
Effects of calcium and magnesium ions on the growth of PaVOA. ***p ≤ 0.001.

The simultaneous addition of calcium and magnesium ions to growing PaVOA had the greatest effect on phage activity (Figure 4), which could significantly improve the titer (*p* < 0.05). The addition of magnesium ions alone could improve the titer (*p* < 0.05), whilst the addition of calcium ions alone would reduce the titer (*p* < 0.05).

#### Phage inactivation test in New Zealand rabbits

**Figure 5:**
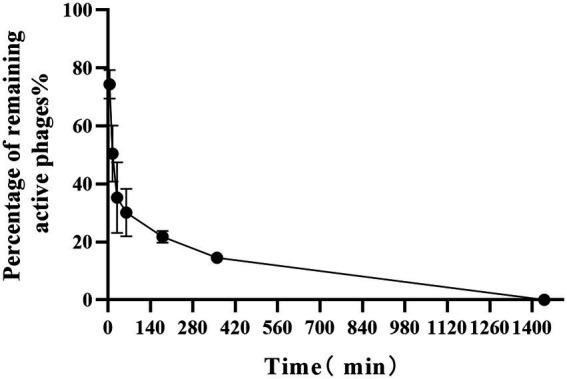
*In vivo* kinetics curve for PaVOA in inoculated New Zealand rabbits.

After the phage was injected into rabbits, phage activity decreased rapidly within 12 h. As shown in Figure 5, phage activity decreased by 25.63 ± 4.88% within 5 min, whilst 50.43 ± 9.68% of the active phage remained after 15 min. An activity of only 14.53 ± 1.38% was observed at 6 h, and no active phages in the blood were observed by 12 h. This indicated that some factors in the blood lead to the inactivation of the PaVOA.

#### Photo documentary and wound healing analyses

**Figure 6:**
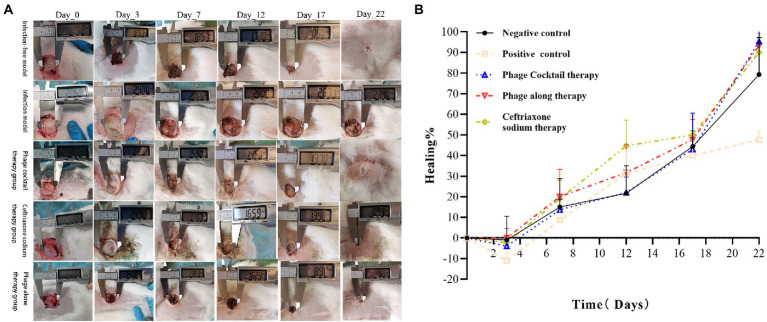
Photo documentary and wound healing. **(A)** Morphological examination of full-thickness excision wounds in New Zealand rabbits by digital photography. **(B)** Percentage of wound healing from day 0 to 22.

The wound healing of the treatment and control groups on days 0, 3, 7, 12, 17, and 22 are shown in Figure 6A. Wound healing percentage results (Figure 6B) showed that the wounds in each group had different degrees of expansion on day 3. From day 12, the wound healing rates of the three infection groups treated with phage cocktail, phage alone, and ceftriaxone were greater than that of the control, with wound healing increases of 22.0%, 31.7%, and 44.7%, respectively. On day 22, the healing rate of the phage cocktail treatment group reached 95.3%, that of the phage alone treatment group reached 93.3%, that of the ceftriaxone treatment group reached 90.0%, and that of the negative control group reached 79.3%, whilst that of the positive control group only reached 47.7%.

#### Histopathological analysis of wound healing

**Figure 7:**
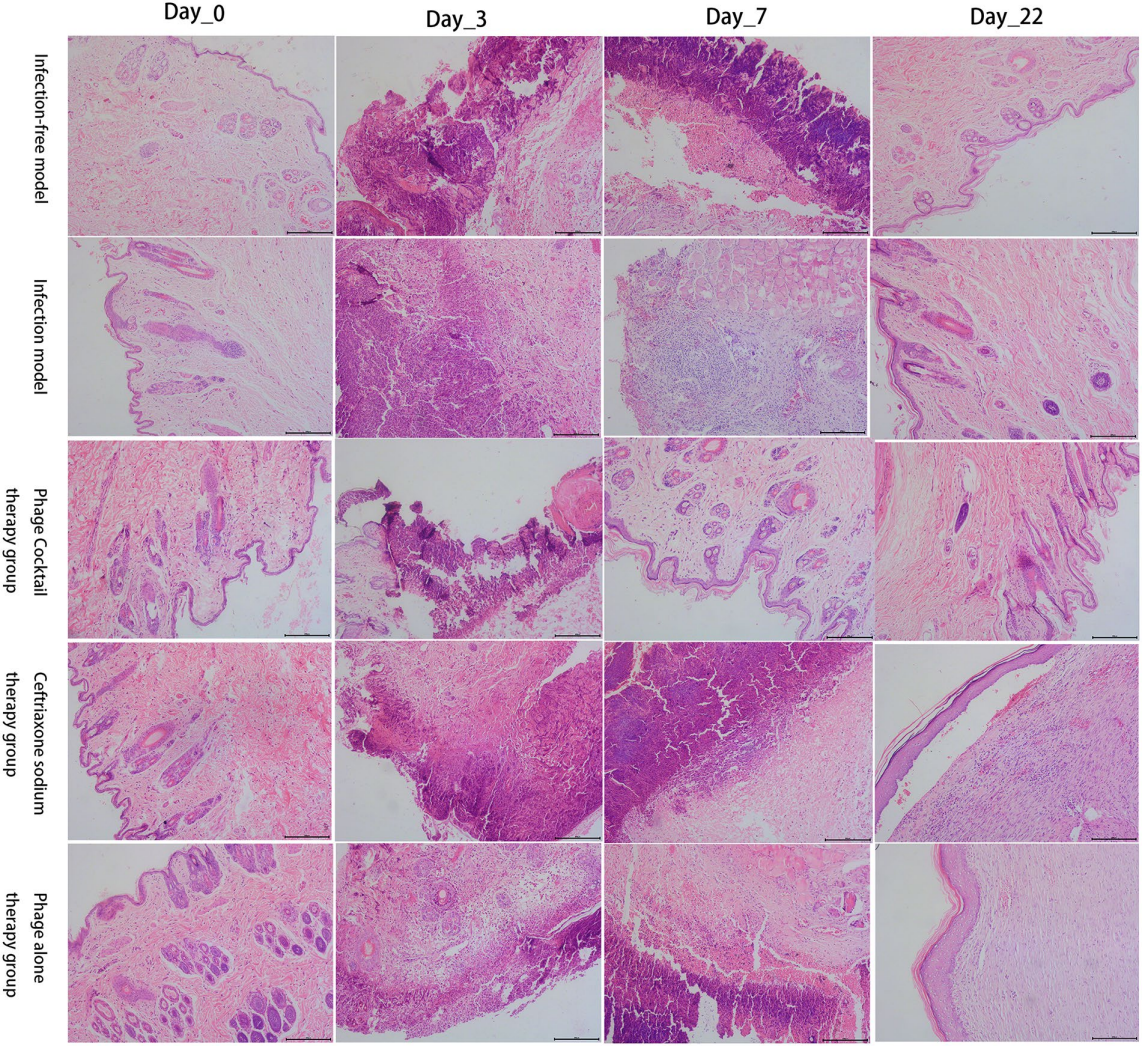
Histological analysis of the healing process of damaged skin in five groups of New Zealand rabbits. Hematoxylin and eosin (H&E) staining (×100).

As shown in Figure 7, all five groups of New Zealand rabbits showed normal skin tissue structures on day 0, with clear epidermal, dermal, and subcutaneous tissue structures. On day 3, after damage and infection, skin sections of the five groups showed varying degrees of structural damage, with a large number of inflammatory exudates and necrotic tissue. At the end of the experiment on day 22, the skin of the negative control (no infection) group and the phage cocktail treatment group had healed completely, and the skin tissue was similar to that observed on day 0. Although the skin of the positive control group (infection without treatment) did not completely heal, skin sections showed good tissue structures. Compared with the results obtained on day 0, there were more sebaceous glands, blood vessels, and other skin appendages distributed, indicating the incomplete maturity of the healing process in the positive control group. Skin sections of the phage alone and ceftriaxone treatment groups showed obvious thickening of the epidermis, fewer skin appendages, and a large number of collagen fibers; however, these are characteristics of the remodeling stage of wound healing. Additionally, the tissue structure of skin slices was relatively clear in the phage cocktail treatment group on day 7, indicating that the wounds were in a stage of rapid healing.

## Discussion

Bacterial infections are a global public health issue (Ahmed and Shimamoto, 2014; Amarillas et al., 2017), and treatment has become more complex as a result of antibiotic abuse and the emergence of MDR bacteria. Phages can accurately kill host bacteria without being limited by bacterial drug resistance and are therefore an ideal means to kill drug-resistant pathogens and pathogenic bacteria with antigenic variation. In this study, the phage PaVOA was isolated from the sewage of Nanfang Hospital in Guangzhou, China. The phage was found to have a dsDNA genome and was able to lyse 100% of the clinical isolates of *P. aeruginosa* strains and produce plaques with a diameter of 2–3 mm. According to plaque morphology and TEM image identification, it is a lytic phage belonging to the *Myoviridae* family.

Notably, phage PaVOA exhibited a narrower host spectrum as compared to phage BrSP1 (de Melo et al., 2019), the latter of which exhibited a lysis rate of 51.4% against 37 *P. aeruginosa* strains, whereas PaVOA only infected 27.6% of the tests strains. Additionally, phage PPaMa1/18 induced an 85.7% lysis of *P. aeruginosa* clinical isolates (Majdani and Shams Ghahfarokhi, 2022). When phages infect bacteria, they mainly bind to host bacterial surface receptors through interactions with the phage RBP (tail spike, tail fiber, and spike protein). As such, host ranges primarily depend on the diversity of phage RBPs. Identifying the RBP of a given phage is helpful to understand the interaction and infection mechanism between the phage and host bacteria. Furthermore, elucidating the structure of phage RBPs opens the possibility of modifying these to further expand the host range of the phage to kill more bacteria.

Studying the biological characteristics of phages will expand the implementation of phages in clinical practice. The optimal MOI of phage PaVOA in this study was 0.1, indicating that PaVOA can infect up to 10 times more bacteria in a small order of magnitude, and can be powerful in practical antibacterial applications. The incubation and outbreak periods of phage PaVOA were 10 and 20 min, respectively. Compared to phage 2019SD1 isolated by Kumar (Kumar et al., 2021) and phage SMP isolated by Ma and Lu (Ma and Lu, 2008), the incubation and outbreak periods of phage PaVOA were shorter, indicating that the phage could infect bacteria more rapidly, thus reflecting the high efficiency of PaVOA. Moreover, during the growth phase of PaVOA, the titer can be increased by the addition of calcium and magnesium ions. It has previously been reported that metal ions play an important role in the adsorption and invasion of phages to host bacteria (Tanji et al., 2004; Moldovan et al., 2007). In this study, magnesium ions significantly promoted phage lysis of bacteria.

The adaptability of phages to their environment is crucial for their clinical application. This study identified that PaVOA activity is relatively stable within the temperature range of −20 to 60°C, with an optimal temperature of 40°C. Overall, these results are conducive to the survival of PaVOA in the environment and clinical setting. PaVOA is more than 60% active in the range of pH 4 to pH 11 and can survive in most acid–base environments. Furthermore, phage PaVOA remained stable for 30 min when exposed to a UV light of 20 W, and was not completely inactivated until after 60 min. These results are similar to results reported by Wang for the stability of phage SLPW (Wang et al., 2016). Overall, measurements of survival at a range of temperatures, pH, and ultraviolet light showed that PaVOA has a strong tolerance to low temperature, extreme pH, and high radiation conditions, which are advantageous characteristics for the clinical application of phages.

In this study, the inactivation of PaVOA in the blood was tested using a New Zealand rabbit model. Phage kinetics showed that at 15 min, the remaining percentage of active phages was 50.43 ± 9.68%. Notably, the rate of decline was fast, and the phage could not be detected in the blood at 12 h. Studies have shown that circulating B cells play an important role in phage inactivation, most likely due to B cells reacting with host immune factors to produce immunoglobulin, resulting in the loss of phage activity (Srivastava et al., 2004). Although this previous study used mice for *in vivo* experiments, we suggest that as both mice and New Zealand rabbits are mammalian species, the overall conclusions from their previous study may also apply to this study. Additionally, phage activity is related to the administration route and phage type, and previous studies reported high anti-phage activity following topical administration, whilst this was less so following oral administration (Lusiak-Szelachowska et al., 2014). In this study, phage inactivation and the absence of animal deaths during local phage treatment also suggested the safety of phage therapy in animals.

Bacterial resistance is constantly increasing, with MDR infections having already caused serious harm to humans. As such, phage therapy is being actively pursued as a substitute for antibiotic therapies in cases involving MDR bacteria. This study selected a strain of MDR *P. aeruginosa* (PA/18) that is resistant to six antibiotics to establish a New Zealand rabbit skin infection model for the comparison of phage (single or combined) and antibiotic (ceftriaxone) therapies for MDR wound infections. Overall, rabbits treated with ceftriaxone exhibited poorer wound healing than those treated with phages. Notably, treatment with a combined phage cocktail resulted in the greatest degree of wound healing. A previous study confirmed that phage cocktail treatment is effective against antibiotic-resistant *Staphylococcus aureus* diabetic foot ulcer infections (Kifelew et al., 2020). It is reported that phage cocktail therapy leads to improved efficacy, with one study suggesting that phage cocktails were able to lyse 86.7% of clinical isolates and that they had synergistic effects when used in combination with antibiotics (Malik et al., 2021). In addition, previous studies have shown that phages, which kill bacteria with minimal side effects (Chadha et al., 2016), may be more effective than antibiotic treatments in wound healing, which is consistent with the findings of this study. Topically applied bacteriophage has been reported to inhibit bacterial biofilm formation and improve wound healing (Mendes et al., 2013; Forti et al., 2018; Yang et al., 2021), which is an advantage of bacteriophage therapy. Regarding histopathological results, accelerated skin healing and remodeling were observed in the phage cocktail treatment group, which was equivalent to the wound healing in the negative control group. Skin regeneration, an indicator for assessing the efficacy of wound treatment, re-epithelization is critical to successful wound healing (Safferling et al., 2013; Rezk et al., 2022). All New Zealand rabbits treated with the phage cocktail in this study exhibited epidermal re-epithelialization and regeneration of skin appendages, similar to New Zealand rabbits before wound infection. In contrast, ceftriaxone-treated New Zealand rabbits had incomplete epidermal formation and lacked skin appendages, similar to the findings of (Rezk et al., 2022). Based on photographic records of wound healing and histopathological analysis, this study demonstrates the potential of phages to treat drug-resistant infections. Additionally, the healing rate in the phage cocktail treatment group was greater than that in the other two treatment groups, which may be attributed to the ability of the phage cocktail to reduce contamination by non-host bacteria (Hooton et al., 2011). In addition, special materials can be selected to carry phage preparations; for instance, using phages embedded in gels to treat infected wounds (Fayez et al., 2021).

## Conclusion

In conclusion, the phage PaVOA discovered in this study belongs to the *Myoviridae* family, is resistant to low temperature, has good tolerance to acid–base and ultraviolet conditions, and is suitable for the environment in clinical treatment. In addition, PaVOA is quickly inactivated in the blood and does not cause animal death. A New Zealand rabbit model of MDR *P. aeruginosa* infection in skin wounds was established, and phage treatment was found to be superior to ceftriaxone treatment. Thus, the phage PaVOA has the potential to treat traumatic skin infections caused by *P. aeruginosa* and may be used as a substitute for antibiotics. With the discovery of new phages, phage cocktail therapy will enter a stage of rapid development for the treatment of MDR bacterial infections.

## Data availability statement

The original contributions presented in the study are included in the article/Supplementary material, further inquiries can be directed to the corresponding author.

## Ethics statement

The animal study was reviewed and approved by Ethics Committee of South China Agricultural University.

## Author contributions

JinyW performed all the experiments and wrote the manuscript. WM, KZ, JingW, BL, and RW participated in the animal experiments. KJ revised the manuscript. All authors contributed to the article and approved the submitted version.

## Funding

This project was supported in part by the Guangdong Province Livestock and Poultry Local Breed Protection and Development and Utilization Promotion Project (2018-143) and Guangdong Province Modern Agricultural Industry Technology System, China (2019kj127).

## Conflict of interest

The authors declare that the research was conducted in the absence of any commercial or financial relationships that could be construed as a potential conflict of interest.

## Publisher’s note

All claims expressed in this article are solely those of the authors and do not necessarily represent those of their affiliated organizations, or those of the publisher, the editors and the reviewers. Any product that may be evaluated in this article, or claim that may be made by its manufacturer, is not guaranteed or endorsed by the publisher.

## References

[ref1] Ahmed A. M. Shimamoto T. (2014). Isolation and molecular characterization of salmonella enterica, Escherichia coli O157:H7 and Shigella spp. from meat and dairy products in Egypt. Int. J. Food Microbiol. 168–169, 57–62. doi: 10.1016/j.ijfoodmicro.2013.10.014, PMID: 24239976

[ref2] Alsaadi A. Beamud B. Easwaran M. Abdelrahman F. El-Shibiny A. Alghoribi M. F. (2021). Learning from mistakes: the role of phages in pandemics. Front. Microbiol. 12:653107. doi: 10.3389/fmicb.2021.653107, PMID: 33815346 PMC8010138

[ref3] Amarillas L. Rubi-Rangel L. Chaidez C. Gonzalez-Robles A. Lightbourn-Rojas L. Leon-Felix J. (2017). Isolation and characterization of phiLLS, a novel phage with potential biocontrol agent against multidrug-resistant *Escherichia coli*. Front. Microbiol. 8:1355. doi: 10.3389/fmicb.2017.01355, PMID: 28785246 PMC5519627

[ref5] Bowler P. G. Duerden B. I. Armstrong D. G. (2001). Wound microbiology and associated approaches to wound management. Clin. Microbiol. Rev. 14, 244–269. doi: 10.1128/CMR.14.2.244-269.2001, PMID: 11292638 PMC88973

[ref6] Breidenstein E. B. de la Fuente-Nunez C. Hancock R. E. (2011). *Pseudomonas aeruginosa*: all roads lead to resistance. Trends Microbiol. 19, 419–426. doi: 10.1016/j.tim.2011.04.005, PMID: 21664819

[ref7] Chadha P. Katare O. P. Chhibber S. (2016). In vivo efficacy of single phage versus phage cocktail in resolving burn wound infection in BALB/c mice. Microb. Pathog. 99, 68–77. doi: 10.1016/j.micpath.2016.08.001, PMID: 27498362

[ref8] Chegini Z. Khoshbayan A. Vesal S. Moradabadi A. Hashemi A. Shariati A. (2021). Bacteriophage therapy for inhibition of multi drug-resistant uropathogenic bacteria: a narrative review. Ann. Clin. Microbiol. Antimicrob. 20:30. doi: 10.1186/s12941-021-00433-y, PMID: 33902597 PMC8077874

[ref9] Chua A. W. Khoo Y. C. Tan B. K. Tan K. C. Foo C. L. Chong S. J. (2016). Skin tissue engineering advances in severe burns: review and therapeutic applications. Burns Trauma 4:3. doi: 10.1186/s41038-016-0027-y, PMID: 27574673 PMC4963933

[ref10] de Melo A. C. C. da Mata Gomes A. Melo F. L. Ardisson-Araujo D. M. P. de Vargas A. P. C. Ely V. L. (2019). Characterization of a bacteriophage with broad host range against strains of *Pseudomonas aeruginosa* isolated from domestic animals. BMC Microbiol. 19:134. doi: 10.1186/s12866-019-1481-z, PMID: 31208333 PMC6580649

[ref11] Dion M. B. Oechslin F. Moineau S. (2020). Phage diversity, genomics and phylogeny. Nat. Rev. Microbiol. 18, 125–138. doi: 10.1038/s41579-019-0311-5 32015529

[ref12] Fayez M. S. Hakim T. A. Agwa M. M. Abdelmoteleb M. Aly R. G. Montaser N. N. (2021). Topically applied bacteriophage to control multi-drug resistant *Klebsiella pneumoniae* infected wound in a rat model. Antibiotics (Basel) 10:1048. doi: 10.3390/antibiotics10091048, PMID: 34572629 PMC8470685

[ref13] Fijan S. Frauwallner A. Langerholc T. Krebs B. Ter Haar Nee Younes J. A. Heschl A. (2019). Efficacy of using probiotics with antagonistic activity against pathogens of wound infections: an integrative review of literature. Biomed. Res. Int. 2019, 7585486–7585421. doi: 10.1155/2019/7585486, PMID: 31915703 PMC6930797

[ref14] Forti F. Roach D. R. Cafora M. Pasini M. E. Horner D. S. Fiscarelli E. V. (2018). Design of a broad-range bacteriophage cocktail that reduces Pseudomonas aeruginosa biofilms and treats acute infections in two animal models. Antimicrob. Agents Chemother. 62, e02573–17. doi: 10.1128/AAC.02573-17, PMID: 29555626 PMC5971607

[ref15] Hooton S. P. Atterbury R. J. Connerton I. F. (2011). Application of a bacteriophage cocktail to reduce salmonella typhimurium U288 contamination on pig skin. Int. J. Food Microbiol. 151, 157–163. doi: 10.1016/j.ijfoodmicro.2011.08.015, PMID: 21899907

[ref16] Kifelew L. G. Warner M. S. Morales S. Vaughan L. Woodman R. Fitridge R. (2020). Efficacy of phage cocktail AB-SA01 therapy in diabetic mouse wound infections caused by multidrug-resistant Staphylococcus aureus. BMC Microbiol. 20:204. doi: 10.1186/s12866-020-01891-8, PMID: 32646376 PMC7346408

[ref17] Krezalek M. A. Alverdy J. C. (2018). The influence of intestinal microbiome on wound healing and infection. Semin. Colon Rectal Surg. 29, 17–20. doi: 10.1053/j.scrs.2017.09.004

[ref18] Kumar P. Meghvansi M. K. Kamboj D. V. (2021). Isolation, phenotypic characterization and comparative genomic analysis of 2019SD1, a polyvalent enterobacteria phage. Sci. Rep. 11:22197. doi: 10.1038/s41598-021-01419-8, PMID: 34772986 PMC8590004

[ref19] Lusiak-Szelachowska M. Zaczek M. Weber-Dabrowska B. Miedzybrodzki R. Klak M. Fortuna W. (2014). Phage neutralization by sera of patients receiving phage therapy. Viral Immunol. 27, 295–304. doi: 10.1089/vim.2013.0128, PMID: 24893003 PMC4076984

[ref20] Ma Y. L. Lu C. P. (2008). Isolation and identification of a bacteriophage capable of infecting Streptococcus suis type 2 strains. Vet. Microbiol. 132, 340–347. doi: 10.1016/j.vetmic.2008.05.013, PMID: 18676101

[ref21] Majdani R. Shams Ghahfarokhi E. (2022). Isolation and characterization of lytic bacteriophages against *Pseudomonas aeruginosa* isolates from human infections in the north-west of Iran. Iran J. Microbiol. 14, 203–213. doi: 10.18502/ijm.v14i2.9189, PMID: 35765555 PMC9168242

[ref22] Malachowa N. McGuinness W. Kobayashi S. D. Porter A. R. Shaia C. Lovaglio J. (2022). Toward optimization of a rabbit model of Staphylococcus aureus (USA300) skin and soft tissue infection. Microbiol. Spectr. 10:e0271621. doi: 10.1128/spectrum.02716-21, PMID: 35389241 PMC9045089

[ref23] Malik S. Nehra K. Rana J. S. (2021). Bacteriophage cocktail and phage antibiotic synergism as promising alternatives to conventional antibiotics for the control of multi-drug-resistant uropathogenic Escherichia coli. Virus Res. 302:198496. doi: 10.1016/j.virusres.2021.198496, PMID: 34182014

[ref24] Mendes J. J. Leandro C. Corte-Real S. Barbosa R. Cavaco-Silva P. Melo-Cristino J. (2013). Wound healing potential of topical bacteriophage therapy on diabetic cutaneous wounds. Wound Repair Regen. 21, 595–603. doi: 10.1111/wrr.12056, PMID: 23755910

[ref25] Moldovan R. Chapman-McQuiston E. Wu X. L. (2007). On kinetics of phage adsorption. Biophys. J. 93, 303–315. doi: 10.1529/biophysj.106.102962, PMID: 17434950 PMC1914437

[ref26] Moradali M. F. Ghods S. Rehm B. H. (2017). *Pseudomonas aeruginosa* lifestyle: A paradigm for adaptation, survival, and persistence. Front. Cell. Infect. Microbiol. 7:39. doi: 10.3389/fcimb.2017.00039, PMID: 28261568 PMC5310132

[ref27] Norbury W. Herndon D. N. Tanksley J. Jeschke M. G. Finnerty C. C. (2016). Infection in burns, Infection in burns. Surg. Infect (Larchmt). 17, 250–255. doi: 10.1089/sur.2013.134, PMID: PMC4790211 26978531

[ref28] Pena C. Cabot G. Gomez-Zorrilla S. Zamorano L. Ocampo-Sosa A. Murillas J. (2015). Influence of virulence genotype and resistance profile in the mortality of Pseudomonas aeruginosa bloodstream infections. Clin. Infect. Dis. 60, 539–548. doi: 10.1093/cid/ciu866, PMID: 25378459

[ref29] Poole K. (2011). Pseudomonas aeruginosa: resistance to the max. Front. Microbiol. 2:65. doi: 10.3389/fmicb.2011.00065, PMID: 21747788 PMC3128976

[ref30] Rathor N. Thakur C. K. Das B. K. Chaudhry R. (2022). An insight into the therapeutic potential of a novel lytic pseudomonas phage isolated from the river ganga. J. Appl. Microbiol. 133, 1353–1362. doi: 10.1111/jam.15639, PMID: 35616159

[ref31] Reynolds D. Kollef M. (2021). The epidemiology and pathogenesis and treatment of Pseudomonas aeruginosa infections: an update. Drugs 81, 2117–2131. doi: 10.1007/s40265-021-01635-6, PMID: 34743315 PMC8572145

[ref32] Rezk N. Abdelsattar A. S. Elzoghby D. Agwa M. M. Abdelmoteleb M. Aly R. G. (2022). Bacteriophage as a potential therapy to control antibiotic-resistant *Pseudomonas aeruginosa* infection through topical application onto a full-thickness wound in a rat model. J. Genet. Eng. Biotechnol. 20:133. doi: 10.1186/s43141-022-00409-1, PMID: 36094767 PMC9468208

[ref33] Sader H. S. Castanheira M. Duncan L. R. Flamm R. K. (2018). Antimicrobial susceptibility of Enterobacteriaceae and *Pseudomonas aeruginosa* isolates from United States medical centers stratified by infection type: results from the international network for optimal resistance monitoring (INFORM) surveillance program, 2015-2016. Diagn. Microbiol. Infect. Dis. 92, 69–74. doi: 10.1016/j.diagmicrobio.2018.04.012, PMID: 29789189

[ref34] Safferling K. Sutterlin T. Westphal K. Ernst C. Breuhahn K. James M. (2013). Wound healing revised: a novel reepithelialization mechanism revealed by in vitro and in silico models. J. Cell Biol. 203, 691–709. doi: 10.1083/jcb.201212020, PMID: 24385489 PMC3840932

[ref35] Shalaby M. Agwa M. Saeed H. Khedr S. M. Morsy O. El-Demellawy M. A. (2019). Fish scale collagen preparation, characterization and its application in wound healing. J. Polym. Environ. 28, 166–178. doi: 10.1007/s10924-019-01594-w

[ref36] Srivastava A. S. Kaido T. Carrier E. (2004). Immunological factors that affect the in vivo fate of T7 phage in the mouse. J. Virol. Methods 115, 99–104. doi: 10.1016/j.jviromet.2003.09.009, PMID: 14656466

[ref37] Stearns-Kurosawa D. J. Osuchowski M. F. Valentine C. Kurosawa S. Remick D. G. (2011). The pathogenesis of sepsis. Annu. Rev. Pathol. 6, 19–48. doi: 10.1146/annurev-pathol-011110-130327, PMID: 20887193 PMC3684427

[ref38] Taati Moghadam M. Khoshbayan A. Chegini Z. Farahani I. Shariati A. (2020). Bacteriophages, a new therapeutic solution for inhibiting multidrug-resistant bacteria causing wound infection: lesson from animal models and clinical trials. Drug Des. Devel. Ther. 14, 1867–1883. doi: 10.2147/DDDT.S251171, PMID: 32523333 PMC7237115

[ref39] Tanji Y. Shimada T. Yoichi M. Miyanaga K. Hori K. Unno H. (2004). Toward rational control of *Escherichia coli* O157:H7 by a phage cocktail. Appl. Microbiol. Biotechnol. 64, 270–274. doi: 10.1007/s00253-003-1438-9, PMID: 13680205

[ref300] Thaden J. T Park L. P. Maskarinec S. A. Ruffin F. Fowler V. G. van Duin D. (2017). Results from a 13-Year prospective cohort study show increased mortality associated with bloodstream infections caused by *Pseudomonas aeruginosa* compared to other bacteria. Antimicrob. Agents Chemother. 61, e02671–16. doi: 10.1128/AAC.02671-16, PMID: 28373189 PMC5444115

[ref40] Wang Z. Zheng P. Ji W. Fu Q. Wang H. Yan Y. (2016). SLPW: a virulent bacteriophage targeting methicillin-resistant Staphylococcus aureus in vitro and in vivo. Front. Microbiol. 7:934. doi: 10.3389/fmicb.2016.00934, PMID: 27379064 PMC4908117

[ref41] Weiner L. M. Webb A. K. Limbago B. Dudeck M. A. Patel J. Kallen A. J. (2016). Antimicrobial-resistant pathogens associated with healthcare-associated infections: summary of data reported to the National Healthcare Safety Network at the Centers for Disease Control and Prevention, 2011-2014. Infect. Control Hosp. Epidemiol. 37, 1288–1301. doi: 10.1017/ice.2016.174, PMID: 27573805 PMC6857725

[ref42] Xu J. Zhang R. Yu X. Zhang X. Liu G. Liu X. (2021). Molecular characteristics of novel phage vB_ShiP-A7 infecting multidrug-resistant Shigella flexneri and Escherichia coli, and its bactericidal effect in vitro and in vivo. Front. Microbiol. 12:698962. doi: 10.3389/fmicb.2021.698962, PMID: 34512574 PMC8427288

[ref43] Yang X. Haque A. Matsuzaki S. Matsumoto T. Nakamura S. (2021). The efficacy of phage therapy in a murine model of *Pseudomonas aeruginosa* pneumonia and sepsis. Front. Microbiol. 12:682255. doi: 10.3389/fmicb.2021.682255, PMID: 34290683 PMC8287650

[ref44] Yoon Kyung Chang R. Nang S. C. Chan H. K. Li J. (2022). Novel antimicrobial agents for combating antibiotic-resistant bacteria. Adv. Drug Deliv. Rev. 187:114378. doi: 10.1016/j.addr.2022.114378, PMID: 35671882

