# Supplementary material for: Topically applied bacteriophage to control multi-drug resistant Pseudomonas aeruginosa-infected wounds in a New Zealand rabbit model
Source: Front Microbiol. 2022 Oct 18;13:1031101. doi: 10.3389/fmicb.2022.1031101 (PMC9624279; doi:10.3389/fmicb.2022.1031101)
Supplement: Supplementary file 1 [file Data_Sheet_1.zip › Supplementary1.docx]

Antibiotic sensitivity of *Pseudomonas aeruginosa*.
